# Survivin regulates intracellular stiffness and extracellular matrix production in vascular smooth muscle cells

**DOI:** 10.1063/5.0157549

**Published:** 2023-10-20

**Authors:** Amanda Krajnik, Erik Nimmer, Joseph A. Brazzo, John C. Biber, Rhonda Drewes, Bat-Ider Tumenbayar, Andra Sullivan, Khanh Pham, Alanna Krug, Yuna Heo, John Kolega, Su-Jin Heo, Kwonmoo Lee, Brian R. Weil, Deok-Ho Kim, Sachin A. Gupte, Yongho Bae

**Affiliations:** 1 Department of Pathology and Anatomical Sciences, Jacobs School of Medicine and Biomedical Sciences, University at Buffalo, Buffalo, New York 14203, USA; 2 Department of Biomedical Engineering, School of Engineering and Applied Sciences, University at Buffalo, Buffalo, New York 14260, USA; 3 Department of Pharmacology and Toxicology, Jacobs School of Medicine and Biomedical Sciences, University at Buffalo, Buffalo, New York 14203, USA; 4 Department of Biochemistry, Jacobs School of Medicine and Biomedical Sciences, University at Buffalo, Buffalo, New York 14203, USA; 5 Department of Orthopedic Surgery, Perelman School of Medicine, University of Pennsylvania, Philadelphia, Pennsylvania 19104, USA; 6 Vascular Biology Program, Boston Children's Hospital, Boston, Massachusetts 02115, USA; 7 Department of Surgery, Harvard Medical School, Boston, Massachusetts 02115, USA; 8 Department of Physiology and Biophysics, Jacobs School of Medicine and Biomedical Sciences, University at Buffalo, Buffalo, New York 14203, USA; 9 Department of Biomedical Engineering, Johns Hopkins University, Baltimore, Maryland 21205, USA; 10 Department of Pharmacology, New York Medical College, Valhalla, New York 10595, USA

## Abstract

Vascular dysfunction is a common cause of cardiovascular diseases characterized by the narrowing and stiffening of arteries, such as atherosclerosis, restenosis, and hypertension. Arterial narrowing results from the aberrant proliferation of vascular smooth muscle cells (VSMCs) and their increased synthesis and deposition of extracellular matrix (ECM) proteins. These, in turn, are modulated by arterial stiffness, but the mechanism for this is not fully understood. We found that survivin is an important regulator of stiffness-mediated ECM synthesis and intracellular stiffness in VSMCs. Whole-transcriptome analysis and cell culture experiments showed that survivin expression is upregulated in injured femoral arteries in mice and in human VSMCs cultured on stiff fibronectin-coated hydrogels. Suppressed expression of survivin in human VSMCs significantly decreased the stiffness-mediated expression of ECM components related to arterial stiffening, such as collagen-I, fibronectin, and lysyl oxidase. By contrast, expression of these ECM proteins was rescued by ectopic expression of survivin in human VSMCs cultured on soft hydrogels. Interestingly, atomic force microscopy analysis showed that suppressed or ectopic expression of survivin decreases or increases intracellular stiffness, respectively. Furthermore, we observed that inhibiting Rac and Rho reduces survivin expression, elucidating a mechanical pathway connecting intracellular tension, mediated by Rac and Rho, to survivin induction. Finally, we found that survivin inhibition decreases FAK phosphorylation, indicating that survivin-dependent intracellular tension feeds back to maintain signaling through FAK. These findings suggest a novel mechanism by which survivin potentially modulates arterial stiffness.

## I. INTRODUCTION

Vascular smooth muscle cells (VSMCs) are a major component of arterial wall structure and comprise the mechanically active cell layer in the tunica media. Vascular injury triggers a phenotypic switch in VSMCs from a differentiated state to a dedifferentiated state. This transition involves increased migration and proliferation as well as the production of large amounts of extracellular matrix (ECM); the ECM forms a structural and mechanical network that plays a key role in regulating both normal and pathological vascular functions.^1–7^ These ECM components, including type 1 collagen (collagen-1), fibronectin, and lysyl oxidase (Lox),^8–12^ undergo significant remodeling in response to vascular injury. These changes, along with vascular calcification, increased oxidative stress and inflammation, and endothelial dysfunction,^13,14^ contribute to arterial stiffening and can lead to neointima formation and thickening,^15–19^ ultimately promoting cardiovascular disease.^20^

Increased cross-linking of collagen-1, a major type of collagen in the tunica media of the arterial wall, strengthens the ECM surrounding VSMCs.^11^ Collagen-1 also binds to integrins linked to biomechanical pathways that affect VSMC migration and proliferation, facilitating the transmission of mechanical forces to VSMCs. Fibronectin, another critical component of the ECM, influences cell–ECM interactions and arterial stiffness by regulating ECM assembly and organization within the arterial wall. Fibronectin also impacts VSMC proliferation through interaction with integrins.^7,21,22^ Similar to collagen-1, inhibiting fibronectin expression reduces cardiac remodeling and fibrosis,^23,24^ potentially contributing to cardiac stiffening.^25^ Lox also promotes VSMC proliferation after vascular injury and during the late stages of atherosclerosis, contributing to abnormal elastin structure^26^ and arterial stiffening in hypertension.^9,27^ The upregulation of Lox expression induces vascular oxidative stress^26^ and triggers arterial stiffening in mouse models.^8^

ECM stiffness is an important mechanical signal for the modulation of cellular function and molecular signaling in VSMCs,^28–33^ and a stiffer microenvironment is associated with cardiovascular disease.^34–37^ Furthermore, mechanical force is essential for ECM interactions,^38,39^ and the interactions between cells and their substrate lead to altered cell morphology, contractility, and stiffness,^40–43^ which are crucial for cell adhesion, migration, proliferation, and survival.^40,44–47^ Mechanical forces from wall shear stress that lead to intimal hyperplasia induce the accumulation of collagen and increase the expression of survivin in VSMCs.^48^ Survivin, also known as baculoviral inhibitor of apoptosis repeat-containing 5 (BIRC5),^49^ is an evolutionarily conserved protein with multi*-*functional roles, acting as both an inhibitor of apoptosis and a regulator of tumor cell proliferation and migration.^50,51^ Its upregulation in cancers often correlates with resistance to various cancer therapies and poor patient prognosis.^52^ Survivin is also upregulated in stiffened arteries under conditions such as vascular injury, atherosclerosis, and hypertension;^53–55^ furthermore, VSMCs derived from stiffened arteries of spontaneously hypertensive rats produce more survivin in addition to the increases in collagen and fibronectin relative to levels from control rats.^56^ Nevertheless, the roles of survivin in VSMCs and its implications in vascular biology and mechanobiology remain poorly understood. We sought to explore how the stiffness of the microenvironment signals changes to ECM production in VSMCs and whether survivin is a regulator of this process.

## II. RESULTS

### A. Genome-wide analysis identifies vascular injury-related expression of ECM components and survivin

**FIG. 1.**
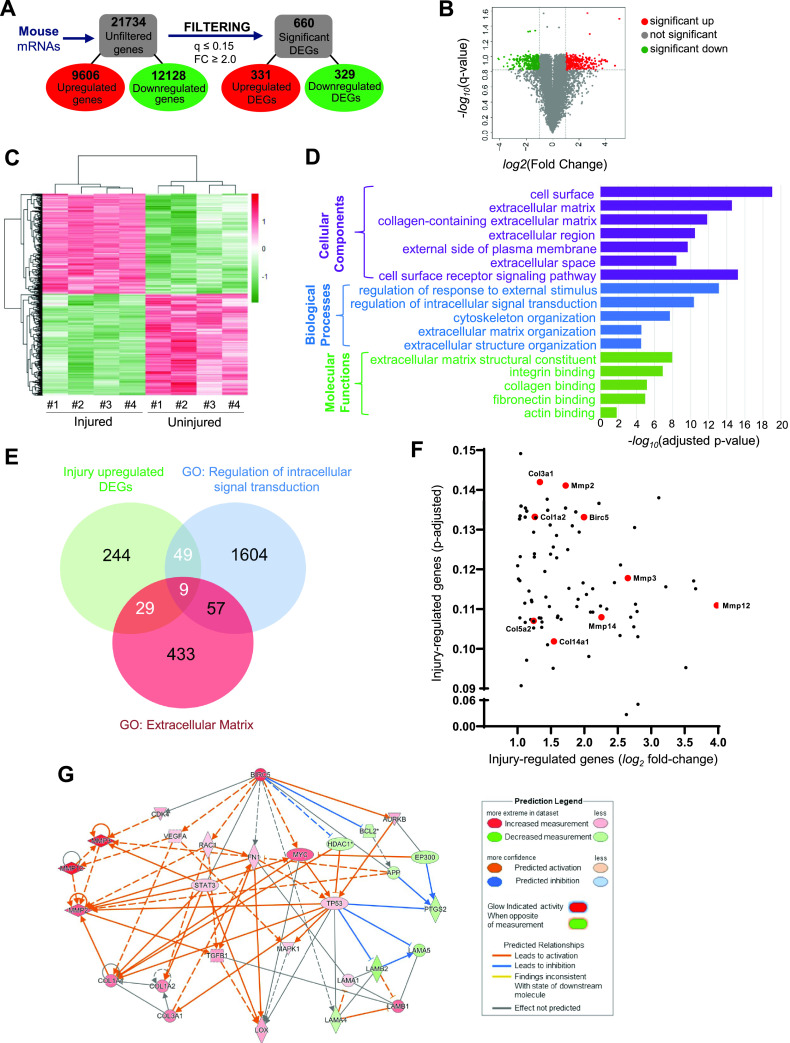
Functional network analysis and predicted network of survivin (*Birc5*) in VSMCs from injured mouse arteries. (a) Reductions in data magnitude by applying significance thresholds to the raw microarray data (fold change [FC] ≥ 2.0, *q* value ≤ 0.15). (b) Volcano plot displays the distributions of all detected genes, represented as single dots that are not statistically different (gray), significantly upregulated (red), or significantly downregulated. (c) Heat map displays the Z-scores of the 660 differentially expressed genes (DEGs). (d) Histogram presenting the significant ECM biological processes (purple), cellular components (blue), and molecular functions^98^ enriched among the 331 DEG upregulated in mouse arteries after injury. (e) Venn diagram of DEGs in mouse vascular injury model. The DEGs upregulated in response to vascular injury (331 genes; green) were compared to GO lists for extracellular matrix (red) and regulators of intracellular signaling transduction (blue). (f) Log_2_ fold-change and adjusted *p* values of the genes positively regulated after vascular injury and contained within the GO terms described above. (g) Network diagram of gene interaction pathways between *Birc5* (survivin) and various ECM proteins, including collagens (*Col1a1*, *Col1a2*, and *Col3a1*), fibronectin (*Fn1*), and lysyl oxidase (*Lox*).

We performed a functional enrichment analysis on differentially expressed genes (DEGs) from previously published microarray data^57^ comparing samples of injured and uninjured mouse femoral arteries. The 21 734 genes in the database were filtered for *q* values of ≤0.15 and fold changes of ≥2.0, revealing a total of 660 DEGs: 331 upregulated and 329 downregulated [Fig. 1(a)]. The distributions of these DEGs against the total number of identified genes were plotted as the *−*log_10_(*q* value) vs log_2_(fold change) values of each detected gene, with red color denoting significantly upregulated DEGs, green denoting significantly downregulated DEGs, and gray denoting genes with no significant change [Fig. 1(b)]. Additionally, the DEGs dataset (Table S1) is depicted in heat maps, showing significant clustering [uninjured vs injured femoral arteries Fig. 1(c)]. To better understand the functional connectivity of our acquired DEGs within our dataset, we further analyzed them based on the Gene Ontology (GO) database using g:Profiler (https://biit.cs.ut.ee/gprofiler). VSMCs at the site of vascular injury in mice were greatly enriched for genes that code for ECM structure and organization/remodeling and cell surface signaling, including those for integrin, collagen, and fibronectin [Fig. 1(d)]. These results prompted us to investigate further the potential mechanism(s) of ECM production in VSMCs. To this end, we analyzed the DEGs that were upregulated after vascular injury and also overlapped with GO terms “extracellular matrix” and “regulation of intracellular signaling transduction.” Among the 331 DEGs upregulated by vascular injury, we identified a total of 87 overlapping genes involved in extracellular matrix proteins and regulators of intracellular signaling [Fig. 1(e)]. The overlapping genes were then plotted to visualize the relative robustness in their expression to vascular injury [Fig. 1(f)]. We observed that, as a group, neither extracellular matrix genes nor mediators of intracellular signaling transduction were strongly regulated during the transcriptomic injury response. Furthermore, the expression of important mediators of ECM structure and reorganization (collagens and MMPs; red dots) were variously distributed within the cluster, suggesting that none had any more functional importance than the others [Fig. 1(f)]. Intriguingly, we found the gene encoding survivin (*Birc5*; red dot) among the regulators of intracellular signaling in the overlapping DEGs [Fig. 1(f)]. *Birc5* has been shown to be upregulated in VSMCs in cardiovascular disease associated with arterial stiffening.^53–55^ Taken together, these findings suggest that *Birc5* may mediate the mechanosensitive response of ECM production in VSMCs.

To identify the relationship among survivin and these DEGs, we used Ingenuity Pathway Analysis (IPA) to build a potential mechanistic network with predictions based on the DEGs. We use the “my pathway” tool to show connections between *Birc5* and all other ECM-related DEGs [Fig. 1(g)]. The network illustrates the associations between *Birc5* and genes for ECM proteins such as collagens (*Col1a1*, *Col1a2*, and *Col3a1*), fibronectin (*Fn1*), and lysyl oxidase (*Lox*) in injured arteries. These analyses demonstrate a potential link between survivin and the ECM in vascular injury that can lead to arterial stiffening.

### B. ECM stiffness modulates survivin expression

**FIG. 2.**
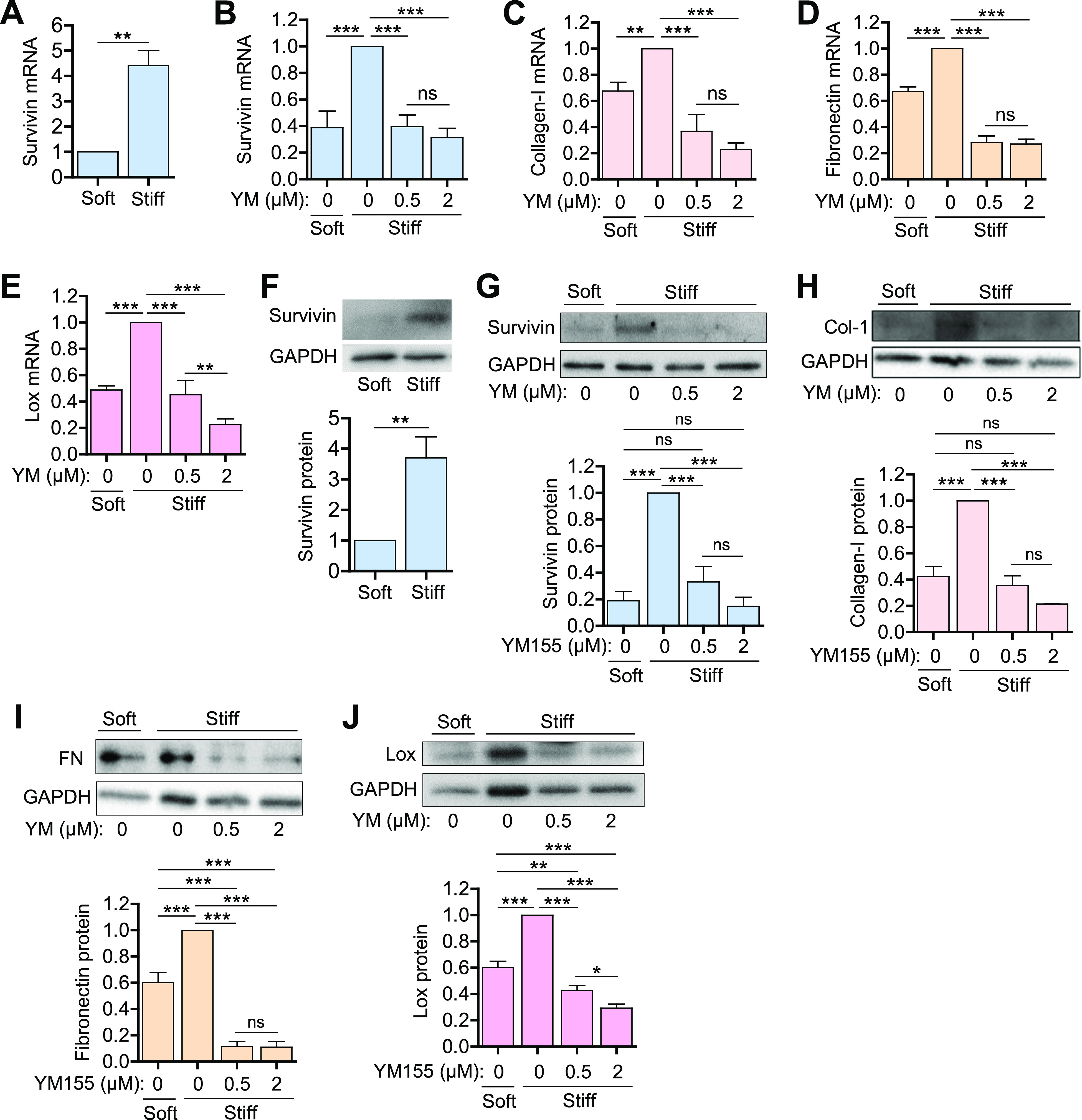
ECM synthesis in hVSMCs is reduced when survivin expression is suppressed. (a) and (f) hVSMCs were synchronized to G_0_ by serum starvation and plated on fibronectin-coated soft or stiff hydrogels with 10% FBS for 24 h. (b)–(e) and (g)–(j) Serum-starved hVSMCs were plated on soft or stiff hydrogels with 10% FBS with DMSO or YM155 at the indicated concentrations for 24 h. Levels of mRNA (a)–(e) and protein (f)−(j) were analyzed by RT-qPCR and immunoblotting assays, respectively. The graphs show the expression of survivin (a), (b), and (e), collagen-I (c) and (h), fibronectin (d) and (i), and Lox (e) and (j). Expression was normalized to that in hVSMCs treated with DMSO (vehicle control) on stiff hydrogels. *n* = 7 (a), *n* = 3 − 8 (b)–(e), *n* = 6 (f), *n* = 4 (g) and (j), *n* = 3 (h) and (i). Error bars show SEMs. ^*^p < 0.05; ^**^p < 0.01; ^***^p < 0.001; and ns, not significant by Student's *t* test (a) and (f) or ANOVA followed by Newman–Keuls post hoc test for multiple comparisons (b)−(e) and (g)–(j).

To investigate the survivin-ECM link, we examined the expression levels of survivin in VSMCs cultured on soft (2–8 kPa) and stiff (16–25 kPa) fibronectin-coated polyacrylamide hydrogels that approximate the physiological stiffness of healthy and diseased vessels, respectively.^57,58^ For these studies, human VSMCs (hVSMCs) were synchronized in G_0_ by serum starvation and plated on the hydrogels with 10% fetal bovine serum (FBS). After 24 h, the cells were collected for reverse transcription-quantitative PCR (RT-qPCR) and immunoblotting assays. Cells grown on stiff hydrogels had increased expression of survivin at the mRNA [Fig. 2(a)] and protein [Fig. 2(f)] levels compared to that in cells grown on the soft hydrogels. hVSMCs plated on stiff hydrogels also had increased expression of three major ECM components, namely, collagen-1 [Figs. 2(c) and 2(h)], fibronectin [Figs. 2(d) and 2(i)], and Lox [Figs. 2(e) and 2(j)].

We also examined the impact of ECM stiffness on ECM deposition on the hydrogels. For this, VSMCs were cultured on both soft and stiff hydrogels for 24 h before they were fixed, stained with phalloidin and fibronectin or collagen, mounted with anti-fade medium containing DAPI, and imaged with a fluorescence microscope. Interestingly, the stiffer substrate promoted the deposition of fibronectin [Fig. S1(a)] and collagen [Fig. S1(b)], indicating that ECM stiffness influences ECM deposition.

To identify the role of survivin in the changes observed in hVSMCs grown on stiff substrates, we treated cells with sepantronium bromide (YM155), which suppresses survivin expression, and measured the expression of several ECM components. YM155 at a concentration of 0.5 *μ*M was sufficient to reduce survivin mRNA and protein levels to those seen in cells grown on the soft substrate [Figs. 2(b) and 2(g)]. Furthermore, YM155 blocked the induction of collagen-I [Figs. 2(c) and 2(h)], fibronectin [Figs. 2(d) and 2(i)], and Lox [Figs. 2(e) and 2(j)], reducing expression to levels below that observed in cells grown on soft substrates.

**FIG. 3.**
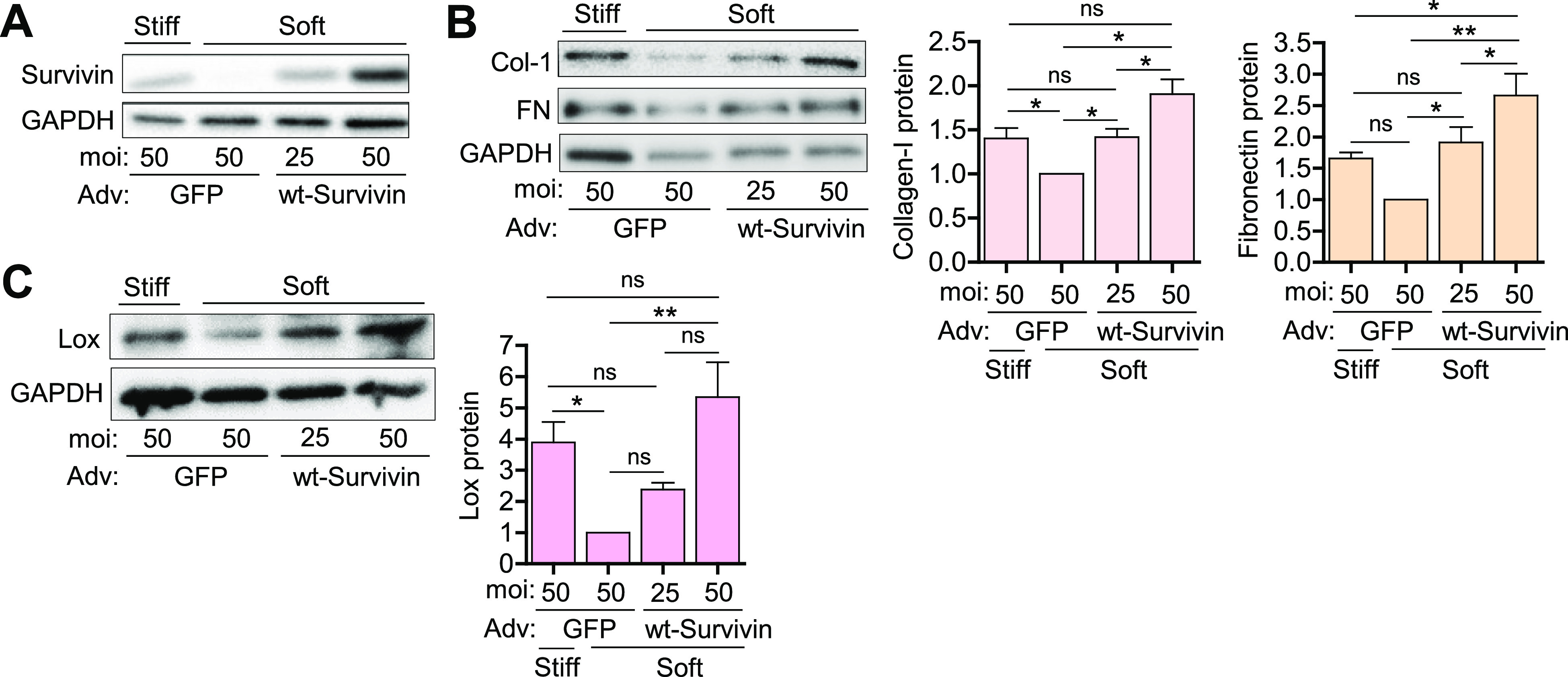
Survivin overexpression mimics stiffness-mediated ECM protein production in hVSMCs. hVSMCs infected with adenoviruses encoding wild-type (wt) survivin or the GFP control were plated on soft or stiff hydrogels with 10% FBS for 24 h. Total cell lysates were analyzed by immunoblotting for survivin (a), collagen and fibronectin (b), and Lox (c). Levels were normalized to those in GFP-expressing hVSMCs on soft hydrogels. *n* = 5 (a), *n* = 3–4 (b), and *n* = 4 (c). Error bars show SEMs. *p < 0.05; **p < 0.01; and ns, not significant by ANOVA followed by Newman–Keuls post hoc test for multiple comparisons.

We next assessed the effect of survivin overexpression. hVSMCs were infected with adenovirus encoding wild-type (wt) survivin (multiplicity of infection [MOI] of 25 or 50) or green fluorescent protein (GFP) as the control (MOI, 50) and plated on soft and stiff hydrogels for 24 h. In hVSMCs cultured on the soft substrate, infection with wt-survivin at an MOI of 25 was sufficient to induce survivin levels to those observed in cells cultured on the stiff substrate, with even greater induction seen at an MOI of 50 [Fig. 3(a)]. Similarly, survivin overexpression induced production of collagen, fibronectin [Fig. 3(b)], and Lox [Fig. 3(c)] to levels observed in cells grown on stiff substrates, with greater induction at the higher MOI. These results show that survivin is sufficient to mimic the ECM production induced when cells are cultured on a stiff substrate. Taken together, the findings suggest that survivin responds to stiffness by coordinating the production of ECM proteins.

### C. ECM stiffness triggers intracellular stiffness via survivin

**FIG. 4.**
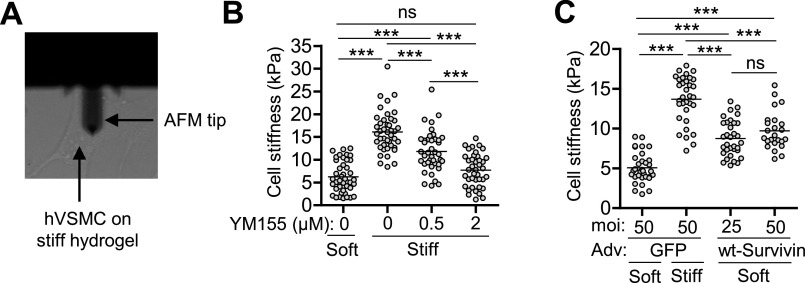
Survivin regulates VSMC stiffness. (a) Visualization of an AFM tip on VSMC surface. hVSMCs were plated on fibronectin-coated soft or stiff hydrogels with 10% FBS for 24 h. Atomic force microscopy was used to measure cellular stiffness in cells treated with YM155 (b) to reduce survivin levels and in cells infected with adenovirus encoding wild-type (wt) survivin or a GFP control for survivin overexpression (c). *n* = 40 − 42 cells from four experiments (b) and *n* = 24–35 cells from three experiments (c). Each data point represents one cell, and the means are indicated by horizontal lines. ***p < 0.001 and ns, not significant by ANOVA followed by Newman–Keuls post hoc test for multiple comparisons.

In addition to increased ECM mRNA and protein production, a stiff substrate affects the mechanical properties of VSMCs, i.e., intracellular stiffness and traction force.^40,59–62^ Thus, we explored the role of survivin in ECM stiffness-mediated changes to intracellular stiffness. We performed atomic force microscopy (AFM) to measure the intracellular stiffness of hVSMCs [Fig. 4(a)] and confirmed that the intracellular stiffness is increased when cells are grown on a stiff substrate, i.e., the stiff fibronectin-coated polyacrylamide hydrogel. This increase in intracellular stiffness was blocked when cells were treated with YM155 to reduce survivin expression [Fig. 4(b)]. Finally, overexpression of survivin was sufficient to increase the stiffness of VSMCs plated on a soft hydrogel [Fig. 4(c)]. These observations indicate that survivin can regulate intracellular stiffness.

### D. Survivin regulates stiffness-mediated Cox2 expression

**FIG. 5.**
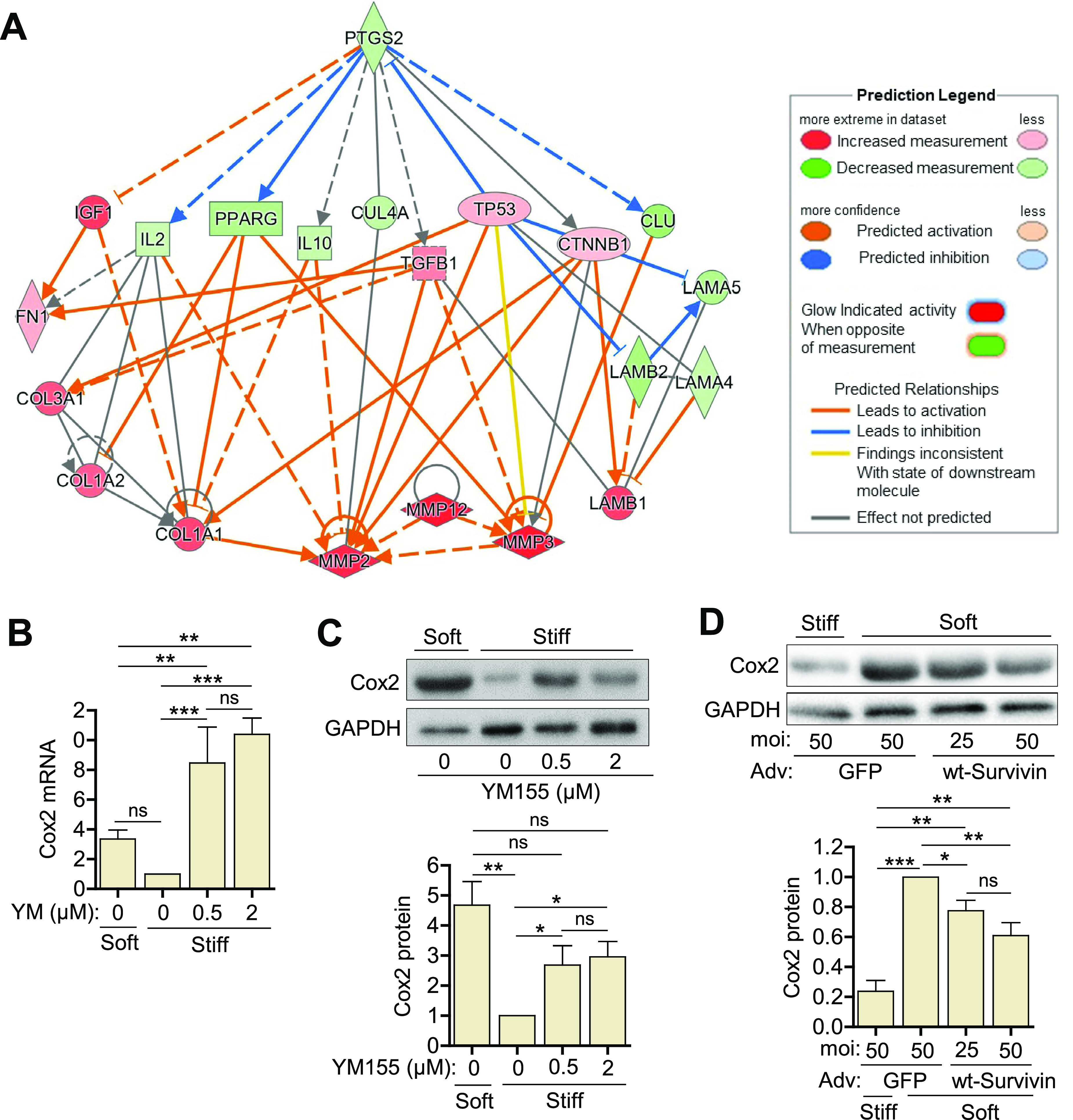
Survivin regulates stiffness-mediated Cox2 expression in hVSMCs. (a) Network diagrams of gene interaction pathways between *Ptgs2* (Cox2) and genes for various ECM proteins. hVSMCs were plated on fibronectin-coated soft or stiff hydrogels with 10% FBS ± YM155 at the indicated concentrations for 24 h. Total cell lysates were analyzed by RT-qPCR (b) and immunoblotting assays (c). hVSMCs infected with adenoviruses encoding GFP or wild-type (wt) survivin were plated on hydrogels with 10% FBS for 24 h. Cell lysates were analyzed by immunoblotting (d). Expression levels were normalized to hVSMCs treated with DMSO (vehicle control) on stiff hydrogels or infected with the virus encoding GFP at an MOI of 50 and plated on soft gels. *n* = 5 (b), *n* = 3 (c) and (d). GAPDH served as a loading control. Error bars show SEMs. *p < 0.05; **p < 0.01; ***p < 0.001; and ns, not significant by ANOVA followed by Newman–Keuls post hoc test for multiple comparisons.

The data presented above indicate that survivin is an important regulator of ECM production and intracellular stiffness in VSMCs. However, it is not yet clear how survivin enacts this regulation. Our previous studies showed that the expression pattern of cyclooxygenase-2 (Cox2), an enzyme involved in prostaglandin biosynthesis, is the inverse of that for ECM proteins in VSMCs on stiff and soft substrates.^63,64^ Therefore, we reasoned that Cox2 signaling may be involved with the regulatory effects of survivin in VSMCs. We used IPA to build a gene interaction network for *Ptgs2*, the gene encoding Cox2 (also known as prostaglandin-endoperoxide synthase 2), and other ECM genes. On the basis of the directionality of the gene interaction arrows and gene expression levels, the network provides evidence that Cox2 contributes to the regulation of ECM proteins [Fig. 5(a)].

We then measured the expression of Cox2 in hVSMCs plated on fibronectin-coated hydrogels. Cox2 was expressed at low levels in cells on stiff substrates, but its expression was markedly upregulated at the mRNA [Fig. 5(b)] and protein [Fig. 5(c)] levels when the cells were treated with YM155 to reduce survivin expression. Furthermore, we found that survivin overexpression decreased Cox2 levels in hVSMCs plated on soft hydrogels [Fig. 5(d)]. Together with the IPA analysis, these results implicate Cox2 in the regulation of ECM proteins and indicate that survivin is a regulator of this function.

### E. Stiffness-mediated survivin expression is regulated by Rac and Rho

Our previous study demonstrated that the ECM stiffness signal is mechanotransduced through the FAK signaling pathway, regulating both VSMC proliferation^57,65^ and survivin.^66^ Additionally, we and others showed that FAK regulates stiffness-mediated Rac activation in VSMCs and that Rac activation promotes VSMC proliferation, intracellular stiffness,^57,65^ and contraction force.^67^ Furthermore, we and others found that Rho activity, another major target of FAK,^68^ is sensitive to ECM stiffness: Rho-GTP levels increase when mouse embryonic fibroblasts^69^ and VSMCs^63^ are plated on stiff hydrogels. Inhibiting Rho activation reduces VSMC stiffness^63^ and contraction force.^70^ ECM stiffness stimulates downstream effectors of Rho—the Rho-kinase (ROCK)-myosin II signaling pathway^71,72^—triggering myosin II-dependent contraction, increased cellular tension, and the formation of actin stress fibers and associated focal adhesions in VSMCs. We therefore investigated whether the inhibition of Rac and Rho affects survivin expression in VSMCs, aiming to uncover a signaling pathway linking intracellular tension (via Rac and Rho) to survivin induction. To explore this, VSMCs infected with adenoviruses encoding LacZ, Rac^N17^ [a dominant negative Rac; Fig. S2(a)], or Rho^N19^ [a dominant negative Rho; Fig. S2(c)] or treated with EHT1864 [a selective Rac inhibitor; Fig. S2(b)], Y27632 [a selective ROCK inhibitor; Fig. S2(d)], or DMSO (control) were cultured on stiff hydrogels for 24 h. Our results show that survivin mRNA levels were reduced when Rho and Rac were inhibited. These findings suggest that intracellular tension is important in regulating survivin expression.

### F. Survivin-dependent intracellular tension feeds back to modulate FAK

**FIG. 6.**
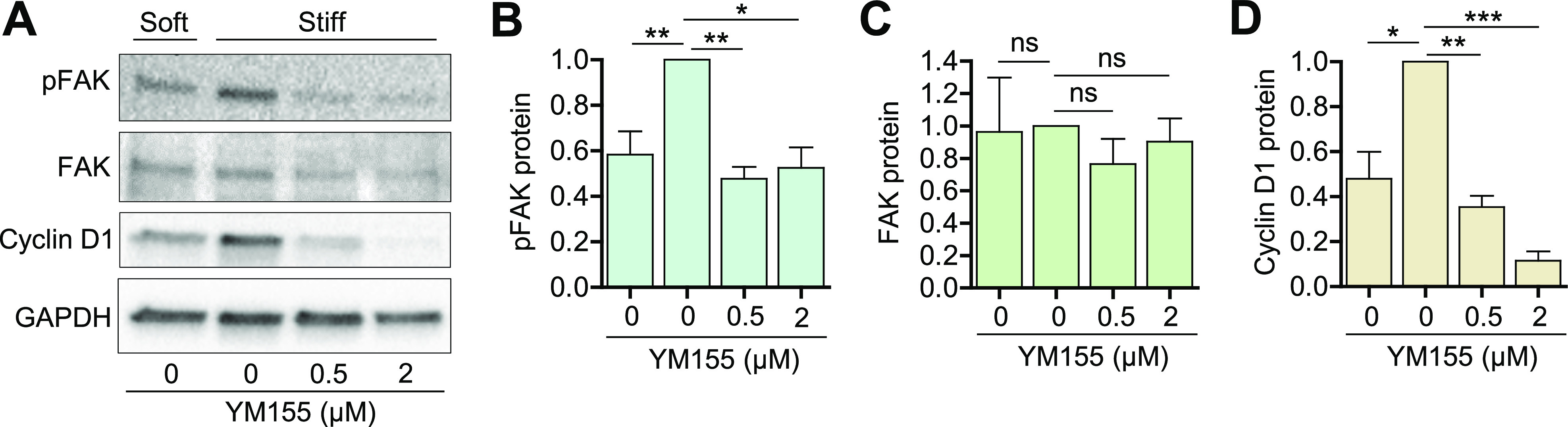
Survivin feeds back to regulate FAK phosphorylation. (a) Serum-starved hVSMCs were plated on fibronectin-coated soft or stiff hydrogels with 10% FBS ± YM155 at the indicated concentrations for 24 h. Total cell lysates were analyzed by immunoblotting for phospho (p)-FAK (b), total FAK (c), and cyclin D1 (d). GAPDH served as a loading control. Expression was normalized to that in hVSMCs treated with DMSO on stiff hydrogels. *n = 3–6*. Error bars show SEMs. *p < 0.05; **p < 0.01; ***p < 0.001; and ns, not significant by Student's *t* test.

FAK plays a key role as an upstream regulator of stiffness-mediated survivin expression and cellular tension. Thus, we asked if inhibiting survivin expression in VSMCs affects FAK activation, as determined by phosphorylation (Fig. 6). To examine this potential feedback loop, VSMCs treated with the survivin inhibitor YM155 or DMSO (control) were cultured on both soft and stiff hydrogels for 24 h. Interestingly, the immunoblotting results demonstrated a significant reduction in FAK phosphorylation [Fig. 6(b)] and cyclin D1 induction [Fig. 6(d)] following the inhibition of survivin. Collectively, these findings along with the data demonstrating that survivin inhibition reduces intracellular stiffness [Fig. 4(b)] indicate that survivin-dependent intracellular tension feeds back to sustain signaling through FAK.

## III. DISCUSSION

Survivin is expressed in response to vascular injury, atherosclerosis, and hypertension in animal models^53,54,73^ and in proliferating VSMCs in the neointima and media in human atherosclerotic plaques and stenotic vein grafts.^54^ However, the role of survivin and the mechanism of its regulation are unknown. We found that survivin expression is sensitive to the stiffness of the ECM and that it induces the production of ECM proteins. Thus, survivin is not only important under conditions associated with arterial stiffening but also possibly exacerbates the stiffening process.

The increased arterial stiffness caused by neointima formation alters the mechanical environment of VSMCs.^74,75^ ECM stiffness and mechanical signals regulate key cellular processes in cardiovascular biology and disease.^76^ Blanc-Brude *et al.*^53^ found that survivin is critical for VSMC survival *in vivo* after acute vascular injury, which is associated with arterial stiffening, suggesting survivin may be stiffness-sensitive.^53^ Our results support this, because survivin was upregulated in cells plated on stiff hydrogels. Our data also suggest that survivin is critical in the transduction of ECM stiffness into intracellular stiffness, which occurs between transmembrane receptors, integrins, and their associated focal adhesion proteins (i.e., FAK), small GTPases (i.e., Rac and Rho), and the actin cytoskeleton.^38,57,69,77–79^

The expression of the wild-type survivin was sufficient to induce ECM production on soft substrates. Furthermore, the stiffness-sensitive induction of ECM proteins was blocked when survivin expression was suppressed by YM155, indicating that survivin may serve as a signal to promote ECM production. These findings are consistent with other studies showing that downregulation of survivin reduces vessel wall thickness and neointima formation in response to vascular injury^80^ and reduces collagen-1 in human Tenon's capsule fibroblasts^49^ and hepatic stellate cells.^81^

We posit that survivin expression stimulates ECM synthesis through two distinct stiffness-dependent pathways: one via Lox activation and the other via Cox2 inhibition. Lox is a collagen crosslinker in the ECM, and its expression paralleled that of survivin. Lox deletion or pharmacological inhibition in mice reduces arterial stiffness and collagen accumulation in atherosclerotic plaques.^64,82,83^ Cox2 also plays a role in cardiovascular biology,^84,85^ possibly contributing to the maintenance of healthy vessels by downregulating collagen and fibronectin synthesis.^63,64^ We found that Cox2 expression is inversely related to both ECM stiffness and survivin expression, and understanding this relationship will give more insight into the molecular processes involved in arterial stiffness. Our data indicate that Cox2 expression is inhibited by survivin and associated with the suppression of ECM production. This new finding is significant, because it may provide a strategy to de-stiffen arteries caused by vascular and cardiovascular diseases.

FAK regulates stiffness-mediated Rac and Rho activation in VSMCs, and Rac and Rho then increase intracellular tension by modulating actin cytoskeletal dynamics and ROCK/myosin II-dependent pathways. Our observations reveal that ECM stiffness and intracellular tension modulate survivin expression, and survivin feeds back to regulate FAK activation, representing both molecular and mechanical pathways through which ECM stiffness regulates survivin. A previous study demonstrated that FAK also modulates YAP,^86^ which is known to stimulate survivin transcription^87^ and is sensitive to ECM stiffness.^88^ Furthermore, several studies showed that YAP signaling is required to maintain cellular tension in various cells.^89–92^ Interestingly, a recent study found that verteporfin, a YAP inhibitor,^93,94^ reduced survivin expression in gastric cancer cells.^95^ Thus, YAP might represent a regulator of survivin to modulate cellular tension.

Overall, the results of this study showed that survivin expression is stiffness-sensitive and that survivin modulates intracellular stiffness and ECM synthesis in VSMCs. These findings provide new insights into the molecular and mechanical mechanisms that control arterial stiffness and VSMC function as well as potential mechano-therapeutic targets for cardiovascular disease.

## IV. METHODS

### A. Cell culture

hVSMCs [Catalog number (Cat. No.) 354-05a, Cell Applications; source: human aorta from a 33-year-old male] were cultured in low-glucose Dulbecco's modified Eagle's medium (DMEM) containing 50 *μ*g/ml gentamicin, 1 mM sodium pyruvate, 1× MEM amino acid solution (Cat. No. M5550, Sigma-Aldrich) and 10% FBS (Cat. No. F2442, Sigma-Aldrich) and used at passages 3–5. hVSMCs were maintained in 10% CO_2_ at 37 °C. Prior to plating on fibronectin-coated (Cat. No. 341631, Calbiochem) polyacrylamide hydrogels, hVSMCs were serum starved for 48 h with DMEM containing 1 mg/ml heat-inactivated, fatty-acid-free bovine serum albumin (BSA; Cat. No. 5217, Tocris) to synchronize their cell cycles to G_0._^57,58^

### B. Preparation of fibronectin-coated polyacrylamide hydrogels

The protocol for generating stiffness-tunable polyacrylamide hydrogels was previously described.^57,96^ Glass coverslips, used as bottom coverslips for hydrogel adhesion, were treated with 0.1 M NaOH solution for 5 min to increase the surface area of the coverslip and enable the subsequently added 3-(trimethoxysilyl)propyl methacrylate (Cat. No. 440159, Sigma-Aldrich) to attach, to which the fibronectin-coated polyacrylamide hydrogels would be covalently linked. The soft hydrogels (2–8 kPa, mimics the physiological stiffness of a healthy mouse^64,69^) and stiff hydrogels (16–25 kPa, mimics the physiological stiffness of a diseased mouse artery^64,69^) were created using various ratios of 40% acrylamide to 1% bis-acrylamide in a solution containing water, 10% ammonium persulfate (Cat. No. A3678, Sigma-Aldrich), TEMED (*N*,*N*,*N*′,*N*′-tetramethylethylenediamine; Cat. No. J63734.AC, Thermo Scientific) to polymerize the solution, and Tris-fibronectin solution consisting of amine-reactive *N*-hydroxysuccinimide ester (Cat. No. A8060, Sigma-Aldrich) dissolved in dimethyl sulfoxide (DMSO; Cat. No. D2650, Sigma-Aldrich) added to 1 M Tris-HCl (pH 8.4) with 0.05% fibronectin. This solution was incubated for 2 h at 37 °C prior to addition into the hydrogel solution; 150 *μ*l of the prepared solution was used for 24- × 24-mm coverslips, 450 *μ*l was used for 24- × 40-mm coverslips, and 20 *μ*l was used for 12-mm coverslips. Glass coverslips were placed on top of the polyacrylamide hydrogels to spread the solution uniformly across the bottom coverslips. To prevent the hydrogel from attaching to the top coverslips, the coverslips were siliconized with 20% Surfasil (Cat. No. TS42801, Thermo Scientific) in chloroform. After polymerization was complete, the top coverslips were removed, and hydrogels were washed three times in 1× Dulbecco's phosphate-buffered saline (DPBS) for 15 min. Hydrogels were blocked for 30 min in serum-free DMEM with 1% BSA prior to cell plating. For different experiments, different-size glass coverslips and plating densities were used: immunoblotting, 24- × 40-mm coverslips with 1 × 10^5^ cells for stiff hydrogels and 2 × 10^5^ cells for soft hydrogels; RT-qPCR and atomic force microscopy, 24- × 24-mm coverslips with 6 × 10^4^ cells for stiff hydrogels and 1.6 × 10^5^ cells for soft hydrogels; immunostaining, 12-mm coverslips with 1.5 × 10^4^ cells for stiff hydrogels and 3 × 10^4^ cells for soft hydrogels.

### C. Cell treatments

#### 1. Pharmacological suppression of survivin expression

hVSMCs were serum starved for 48 h with DMEM containing 1 mg/ml BSA and then plated on fibronectin-coated hydrogels for 24 h in DMEM with 10% FBS containing 0.5 *μ*M or 2.0 *μ*M YM155 (an inhibitor of survivin promoter activity; Cat. No. 6491, Tocris), 30 *μ*M Y27632 (a selective ROCK inhibitor), or 20 *μ*M EHT1864 (an inhibitor of Rac family GTPases) in DMSO.

#### 2. Adenovirus infection

To overexpress survivin, hVSMCs were incubated in DMEM with 1 mg/ml BSA for ∼7 h, and adenovirus harboring *BIRC5* (Cat. No. 1611, Vector Biolabs) or the gene for GFP (experimental control; Cat. No. 1060, Vector Biolabs) was added to the medium at an MOI of 25 or 50. Additionally, Rho^N19^ (a dominant negative Rho) at 300, Rac^N17^ (a dominant negative Rac) at 100, and LacZ (experimental control) at 100 or 300 were gifted from Dr. Richard Assoian (University of Pennsylvania) and were used. Cells were serum starved for an additional 40 h before they were plated on fibronectin-coated hydrogels with DMEM containing 10% FBS for 24 h.

### D. RNA isolation and RT-qPCR

Cells cultured on hydrogels were washed twice with 1× DPBS, and RNA was extracted with TRIzol as described by Thermo Fisher's TRIzol RNA extraction protocol.^97^ A NanoDrop Lite spectrophotometer (Cat. No. ND-LITE-PR, Thermo Scientific) was used to determine RNA purity and concentration. Total RNA was reverse transcribed and analyzed by RT-qPCR as previously described.^97^ TaqMan probes (Invitrogen) for hVSMCs were used for survivin (*BIRC5;* Hs04194392_m1), collagen-1A1 (*COL1A1*; Hs00164004_m1), fibronectin-1 (*FN1*; Hs01549976_m1), Lox (*LOX*; Hs00942480_m1), Cox2 (*PTGS2*; Hs00153133_m1), and *GAPDH* (Hs02786624_g1). The comparative cycle threshold method was used to determine the mRNA expression for each target gene using the gene for GAPDH as the reference.

### E. Protein extraction and immunoblotting

hVSMCs cultured on hydrogels were washed twice with cold 1× DPBS. The glass coverslips supporting the hydrogels were placed face-down on 150 *μ*l of 5× sample buffer (250 mM Tris-HCl [pH 6.8], 10% sodium dodecyl sulfate, 50% glycerol, 0.02% bromophenol blue, and 10 mM 2-mercaptoethanol) and incubated for 2 min at room temperature to obtain total cell lysates.^57,58,96^ The proteins in the resulting total cell lysates were denatured at 100 °C, subjected to 6%–12% sodium dodecyl sulfate-polyacrylamide gel electrophoresis, and transferred electrophoretically onto polyvinylidene difluoride membranes. The membranes were blocked with 6% milk in 1× Tris-buffered saline with 0.1% Tween 20 (TBST) for 1.5 h before they were incubated overnight at 4 °C with antibodies to survivin (Cat. No. NB500-201, Novus Biologicals), collagen (Cat. No. C2456, Sigma-Aldrich), fibronectin (Cat. No. F3648, Sigma-Aldrich), Cox2 (Cat. No. 66351-1-Ig, Proteintech), Lox (Cat. No. NB100-2530, Novus Biologicals), phospho-FAK (Cat. No. 3283, Cell Signaling Technology), FAK (Cat. No. ZF002, Thermo Fisher Scientific), cyclin D1 (Cat. No. sc-20044, Santa Cruz Biotechnology), and GAPDH (10494-1-AP, Proteintech). After overnight incubation at 4 °C, membranes were washed with 1× TBST for 15 min and probed with secondary antibody for 1 h at room temperature. Blots were washed with 1× TBST for 15 min before imaging. Clarity Western ECL substrate (Cat. No. 1705061, Bio-Rad) or Clarity Max Western ECL substrate (Cat. No. 1705062, Bio-Rad) were used for antibody detection.

### F. Immunostaining

hVSMCs cultured on hydrogels for 24 h were fixed with 3.7% formaldehyde in DPBS for 1.5 h, permeabilized with 0.4% Triton X-100 for 1 h, and then blocked with 2% BSA and 0.2% Triton X-100 in DPBS for 1 h at room temperature (RT). Subsequently, cells were then incubated with a 1:400 dilution of anti-fibronectin antibody (Cat. No. F3648, Sigma-Aldrich) or a 1:100 dilution of anti-collagen 1A1 antibody (Cat. No. E8I9Z, Cell Signaling Technology) for 1 h at RT, followed by three washes with DPBS containing 2% BSA and 0.2% Triton X-100. Next, cells were incubated with a 1:200 dilution of Alexa Fluor 594 goat anti-rabbit IgG (Cat. No. A11037, Invitrogen) and a 1:200 dilution of Alexa Fluor 488 phalloidin (Cat. No. A12379, Invitrogen) for 1h at RT. Afterward, cells were washed three times with DPBS containing 2% BSA and 0.2% Triton X-100 and then washed once with distilled water. Subsequently, cells were mounted with DAPI-containing mounting medium (Cat. No. P36962, Thermo Fisher Scientific) on microscope slides. Fluorescence images of cells were captured using Cytation 1 Imaging Reader (Agilent Technologies) with 20× objective. For each sample, 10–15 fields of view were taken to examine ECM synthesis and deposition. For each set, settings for exposure, integration time, and camera gain were maintained constant for accurate comparison between samples.

### G. Atomic force microscopy

Atomic force microscopy was used to measure the intracellular stiffness of cells as described previously.^57,58^ The surfaces of the cells cultured on hydrogels were indented with a silicon nitride cantilever (Cat. No. BL-AC40TS-C2; Asylum; spring constant, 0.09 N/m) with a three-sided pyramidal tip (8-nm in radius). The stiffness of each cell was measured in contact mode using an NX12 AFM system (Park Systems) mounted on a Nikon ECLIPSE Ti2 inverted microscope. To analyze the stiffness, the first 400 nm of horizontal tip deflection was fit with the Hertz model for a three-sided pyramid and a 35° face angle. For each experimental condition, three force curves per cell were acquired for a total of 10 cells under each condition. Measurements were taken at three evenly spaced locations on the cell membrane. Atomic force microscopy experiments were independently repeated three times (obtaining a total of 30 force curves for each experimental condition). Using atomic force microscopy analysis software XEI (Park Systems), the force curves were quantified and converted to Young's modulus (stiffness).

### H. Functional network analysis

(i) Gene expression analysis: differential gene expression analysis was previously performed on microarray data.^57^ Duplicate and blank (no name) gene entries were removed, and genes with insignificant differential expression values were filtered out before further analysis. DEGs were defined as those having a fold-change of ≥2.0 and a *q* value of ≤0.15. The R programming language's heatmap package and Python's bioinfokit package were used to create a heatmap and volcano plot, respectively. The heatmap was created using normalized count data with an unsupervised approach. The Euclidean distance was used to cluster samples based on the complete method.
(ii) Functional enrichment analysis: functional enrichment analysis was performed using the g:GOSt tool in gProfiler (https://biit.cs.ut.ee/gprofiler/gost). The statistical domain scope of the analysis was only annotated genes, and the significance threshold was set to the g:SCS algorithm for computing multiple-testing corrections for *p* values acquired from Gene Ontology (GO) analysis. Significant GO terms were defined by an adjusted *p* value of ≤0.05. Biological GO annotations from each of the three main GO categories (Biological Processes, Cellular Components, and Molecular Functions) were considered for this analysis along with those deemed relevant to ECM or mechanosensitive signaling activity. Process GO terms were presented in histograms on a scale of −log(adjusted *p* value). The Venn diagram in Fig. 1(e) was generated using the DEGs output dataset, with a cutoff of 2.0 log_2_ positive fold-change with an adjusted q-value of <0.15. The upregulated genes resulting from vascular injury were compared to GO gene lists for extracellular matrix and regulation of intracellular signal transduction (GO terms 0031012 and 1902531, respectively).

### I. Network analysis

IPA (Qiagen) was used to perform further bioinformatics analysis on the filtered microarray data. A Core Analysis was run, which returned information on various mechanistic pathways and enriched functions on the basis of the literature compiled in the Ingenuity Knowledge Base. The “Diseases and Functions” tool was used to identify molecules known to be involved in ECM synthesis within the microarray dataset, and the “My Pathway” tool was subsequently used to display known relationships between *Birc5* and other genes within the ECM synthesis function. The z-directional components of the expression analysis were based on the expression log ratio values. Functions with a z-score of >2 were regarded to have significant activation, whereas those with a z-score of <−2 were considered to have significant inhibition. The “Molecule Activity Predictor” tool was used to display gene expression levels by node color and intensity and to generate predicted activation states of molecules and interactions based on results of the Core Analysis.

### J. Statistical analysis

Statistical significance was determined using Prism (Graph-Pad) software. Data are presented as means and standard errors of the means (SEMs) and were analyzed with Student's *t* tests or ANOVAs followed by Newman–Keuls post hoc test for multiple comparisons as appropriate. Samples sizes for each group are indicated in the figure legends.

## SUPPLEMENTARY MATERIAL

See the supplementary material for one table with microarray data.

## Data Availability

The data that support the findings of this study are available from the corresponding author upon reasonable request.
